# Biological role of miR-204 and miR-211 in melanoma

**DOI:** 10.18632/oncoscience.443

**Published:** 2018-08-22

**Authors:** Marianna Vitiello, Romina D’Aurizio, Laura Poliseno

**Affiliations:** ^1^ Oncogenomics Unit, CRL-ISPRO, 56124 Pisa, Italy; ^2^ Institute of Clinical Physiology, CNR, 56124 Pisa, Italy; ^3^ Institute of Informatics and Telematics, CNR, 56124 Pisa, Italy

**Keywords:** melanoma, small RNA-seq, BRAF inhibitors, miR-204, miR-211

## Abstract

In this short report, we pinpoint some technical and conceptual flaws that we found in the article entitled “miR-204-5p and miR-211-5p contribute to BRAF inhibitor resistance in melanoma” (Díaz-Martínez et al., Cancer Research 2018). We also discuss how, in our opinion, these flaws led Díaz-Martínez and colleagues to incorrect conclusions about the biological role that miR-204 and miR-211 play in melanoma and about the terms of their involvement in the phenomenon of resistance to BRAF inhibitors.

## REPORT

With the aim to identify the microRNAs involved in resistance to vemurafenib, in the research article entitled *“miR-204-5p and miR-211-5p contribute to BRAF inhibitor resistance in melanoma”* Díaz-Martínez and colleagues performed small RNA sequencing on A375 parental cells and the resistant A375-VR population, looking for differentially expressed microRNAs [1].

miR-204 was chosen because its levels are ~2- fold higher in A375-VR vs A375, as detected by small RNA-seq and confirmed by qRT-PCR. Consistently with our previously published data (Vitiello et al., *Context- dependent miR-204 and miR-211 affect the biological properties of amelanotic and melanotic melanoma cells*, Oncotarget [2]), Díaz-Martínez and colleagues show that in A375 cells (but not in A375-VR cells) the ERK pathway negatively regulates miR-204. They also claim that miR-204 is positively involved in resistance to vemurafenib. However, this claim is formally supported only by the mild decrease in proliferation that A375-VR show when transfected with a miR-204 inhibitor and exposed to vemurafenib (Figure 5E in reference 1) [3,4]. Conversely, we and others have extensively demonstrated both *in vitro* and using patient data that miR-204 rather exerts its activity in sensitive cells, where its induction upon vemurafenib treatment is very robust, it targets AP1S2 (a validated pro-motility target not considered by Díaz-Martínez and colleagues) and it potentiates the anti-motility effects of the drug, in turn behaving as an oncosuppressor [2, 5].

**Figure 1:**
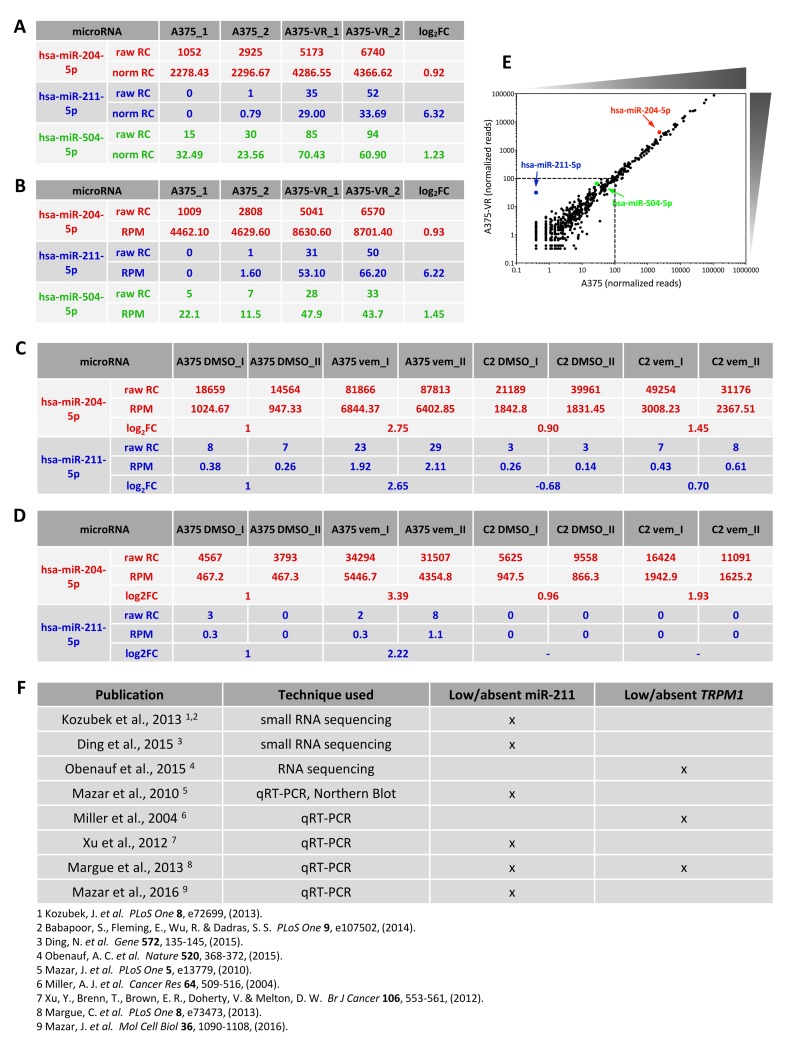
Low expression levels of miR-211 in A375 cells **(A-D)** Expression levels of miR-204 and miR-211, as detected by small RNA sequencing in Díaz-Martínez et al., 2018 **(A-B)** and in our paper (Vitiello et al., 2017, **(C-D)**). In A, the raw and normalized read counts (RC) of miR-204 and miR-211 are listed, as available at GSE107576. The log2FC reported in Supplementary Table 2 of Díaz-Martínez et al., 2018 are shown as well. In B, the raw read counts of miR-204 and miR-211 were recalculated by us, starting from the raw reads available at GSE107576 and following the analytical steps described in Vitiello et al., 2017. For consistency with the analysis performed by Díaz-Martínez and colleagues, the match with known microRNAs (miRBase v.21) was subjected to 100% identity. The reads per million (RPM) and the log2FC are shown as well. In C, the raw read counts of miR-204 and miR-211 are listed, as available at GSE94423. The RPM and the log2FC reported in Vitiello et al., 2017 are shown as well. In order to better compare our data with the data produced by Díaz-Martínez and colleagues, in D we recalculated the raw read counts and RPM of miR-204 and miR-211, starting from the raw reads available at GSE94423 and following the same analytical steps as in B. In both datasets, miR-204 and miR-211 show higher expression level in the resistant cells (A375-VR and A375 C2 vem) compared to A375 parental cells. However, the layout of the sequencing performed by us allows to appreciate that the most profound increase is the one shown by both microRNAs in A375 parental cells upon vemurafenib treatment. Furthermore, both datasets indicate that miR-211 is expressed at very low level, much lower than that of miR-204 and even lower that that of miR-504, which Díaz-Martínez and colleagues did not prioritize for further analysis on the basis of this very reason. Contrary to Díaz- Martínez and colleagues, we decided to apply a threshold and consider only the microRNAs that we found expressed at > 100 reads in at least one experimental condition. Accordingly, we discarded miR-211 and focused only on miR-204. The depths of the 2 small RNA sequencing are the following: Díaz-Martínez et al., 2018: 7.7million reads per sample on average; Vitiello et al., 2017: 23.3 million reads per sample on average. **(E)** Dot plot of the normalized reads of the microRNAs identified in A375 cells (x axis) vs A375-VR cells (y axis) in Díaz-Martínez et al., 2018. The graph highlights that the distribution of microRNA expression levels in the two cell lines is overall very similar. It also shows that miR-504, and even more miR-211, belong to the tail of low expressed and highly scattered microRNAs (<100 normalized reads). **(F)** List of additional publications in which the expression of *TRPM1*/miR-211 has been analyzed in A375 cells and found to be very low (much lower than that of *TRPM3*/miR-204) or even absent.

miR-211, the other member of the same microRNA family, was also prioritized in light of its higher expression level in A375-VR vs A375 (~80-fold according to small RNA-seq, ~2-fold according to qRT-PCR) [1]. Since in A375 cells the basal levels of miR-211 are substantially lower than those of miR-204 (please refer to Figure 1 and its caption for details about the analysis of microRNA expression levels), these data raise multiple concerns.

First, it is unclear why the authors discarded miR- 504 due to its low expression levels and yet they went after miR-211 that is expressed even less (Figure 1).

**Figure 2:**
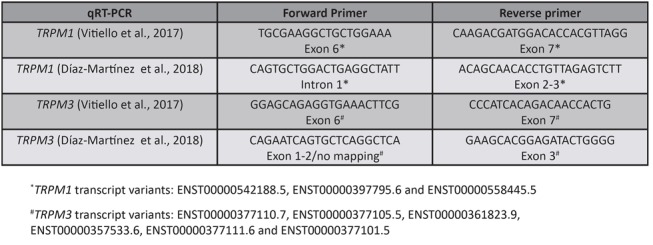
Location of qRT-PCR primers used to detect *TRPM1* (miR-211 host gene) and *TRPM3* (miR-204 host gene) For the detection of *TRPM1* and *TRPM3*, we chose primers that are located in E6 and E7, which are the exons that flank the intron from which the microRNAs are expressed, while Díaz-Martínez and colleagues did not. In light of the fact that host genes are characterized by multiple isoforms, this strategy is considered the most accurate when the expression level of the mature microRNA and that of its host gene need to be correlated (Mikhaylova et al., 2012^1^). For a more detailed description of the location of *TRPM1* and *TRPM3* qRT-PCR primers, please refer to Supplementary information. ^1^Mikhaylova O, Stratton Y, Hall D, Kellner E, Ehmer B, Drew AF, Gallo CA, Plas DR, Biesiada J, Meller J and Czyzyk- Krzeska MF. VHL-regulated MiR-204 suppresses tumor growth through inhibition of LC3B-mediated autophagy in renal clear cell carcinoma. Cancer Cell. 2012; 21(4):532-546.

Second, the accuracy of mature miR-211 detection by qRT-PCR is questionable. No evidence is provided about the specificity of the Taqman probes used, in spite of the fact that miR-211 is very similar in sequence to miR-204. In addition, the location of the primers used for the qRT-PCR detection of *TRPM1* host gene is suboptimal (Figure 2). miR-204 is likely detected instead of or together with miR-211 and this is why they both show a ~2-fold increase in expression according to qRT-PCR.

Finally, we question the biological relevance of a microRNA that is still expressed at very low levels even when upregulated. Since they belong to the same family, it is not surprising that miR-211 behaves like miR-204, if exogenously overexpressed [1, 2]. However, we and others have shown that the appropriate biological context to study endogenous miR-211 are not amelanotic cells, like A375 cells, but melanotic ones: only there miR-211 shows high basal levels (actually higher than those of miR-204) and is able to limit the efficacy of vemurafenib, by exerting its MITF-dependent pro-pigmentation activity [2, 6, 7].

## SUPPLEMENTARY MATERIALS


