# Supplementary material for: Biological role of miR-204 and miR-211 in melanoma
Source: Oncoscience. 2018 Aug 22;5(7-8):248–51. doi: 10.18632/oncoscience.443 (PMC6142896; doi:10.18632/oncoscience.443)
Supplement: Supplementary file 1 [file oncoscience-05-248-s001.pdf]

SUPPLEMENTARY MATERIALS

Location of *TRPM1* qRT-PCR primers

According to Ensembl Genome Browser, seven protein-coding transcript variants of *TRPM1* exist: ENST00000542188.5, ENST00000397795.6, ENST00000256552.10, ENST00000559179.1, ENST00000558768.5, ENST00000558445.5 and ENST00000559177.5.

TRPM1 forward and reverse primers (TGCGAAGGCTGCTGGAAA and CAAGACGATGGACACCACGTTAGG, Vitiello et al., 2017) align with exon 6 and exon 7 of ENST00000542188.5, ENST00000397795.6 and ENST00000558445.5. They also align with exon 7 and exon 8 of ENST00000256552.10, as well as with exon 3 and exon 4 of ENST00000558768.5. In all the cases listed above, the exons involved are those that surround the intron in which miR-211 gene is located. In ENST00000559177.5, the primers align with intron 4, where miR-211 gene is also located. Conversely, they do not align to ENST00000559179.1, a variant from which miR-211 is not processed.

TRPM1 forward and reverse primers (CAGTGCTGGACTGAGGCTATT and ACAGCAACACCTGTTAGAGTCTT, Díaz-Martínez et al., 2018) align with intron 1 and exon 2-3 junction of ENST00000542188.5, ENST00000397795.6, ENST00000559179.1, ENST00000558445.5 and ENST00000559177.5. They also align with exon 2 and exon 3-4 junction of ENST00000256552.10. They do not align with ENST00000558768.5.

Location of *TRPM3* qRT-PCR primers

According to Ensembl Genome Browser, fourteen transcript variants of *TRPM3* exist: ENST00000377110.7, ENST00000360823.6, ENST00000377105.5, ENST00000396292.8, ENST00000358082.7, ENST00000408909.6, ENST00000396280.9, ENST00000361823.9, ENST00000357533.6, ENST00000396285.5, ENST00000377111.6, ENST00000396283.5, ENST00000377101.5 and ENST00000377097.3.

TRPM3 forward and reverse primers (GGAGCAGAGGTGAAACTTCG and CCCATCACAGACAACCACTG; Vitiello et al., 2017) align with: exon 6 and exon 7 of ENST00000377110.7, ENST00000377105.5, ENST00000361823.9, ENST00000357533.6, ENST00000377111.6 and ENST00000377101.5; exon 6 and exon 8 of ENST00000360823.6 and ENST00000396283.5; exon 4 and exon 6 of ENST00000396292.8 and ENST00000358082.7; exon 4 and exon 5 of ENST00000408909.6, ENST00000396280.9 and ENST00000396285.5. In all the cases, the exons involved surround the intron in which miR-204 gene is located.

TRPM3 forward and reverse primers (CAGAATCAGTGCTCAGGCTCA, GAAGCACGGAGATACTGGGG, Díaz-Martínez et al., 2018) align with exon 1-2 junction and exon 3 of ENST00000361823.9, ENST00000396283.5 and ENST00000377101.5. The reverse primer aligns with exon 3 also in ENST00000377110.7, ENST00000377105.5, ENST00000360823.6, ENST00000357533.6 and ENST00000377111.6. Furthermore it aligns with the 5' upstream sequence of ENST00000396292.8, ENST00000358082.7 and ENST00000408909.6, ENST00000396280.9 and ENST00000396285.5. However, in these transcripts there is no mapping for *TRPM3* forward primer.

In ENST00000377097.3, there is no mapping for any of the 4 primers.
